# Supplementary material for: A mathematical model of ephaptic interactions in neuronal fiber pathways: Could there be more than transmission along the tracts?
Source: Netw Neurosci. 2020 Jul 1;4(3):595–610. doi: 10.1162/netn_a_00134 (PMC7462434; doi:10.1162/netn_a_00134)
Supplement: Supplementary file 1 [file netn-04-595-s001.pdf]

Sheheitli, H., & Jirsa, V. K. (2020). Supporting information for "A mathematical model of ephaptic interactions in neuronal fiber pathways: could there be more than transmission along the tracts?" *Network Neuroscience*, 4(3), 595–610.
https://doi.org/10.1162/netn_a_00134

A mathematical model of ephaptic interactions in neuronal fiber pathways: could there be more than transmission along the tracts?

Hiba Sheheitli, Viktor K.Jirsa

1 Supporting information

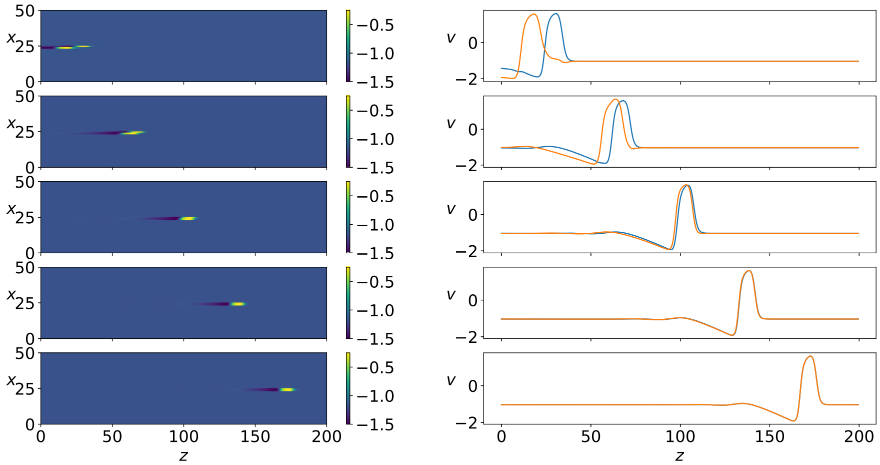

Figure 1: **(SI Fig.1) Shape of impulses along axons.** Two adjacent impulses starting out with small enough phase difference get attracted to each other and move in phase. Same as in Fig.3(b)

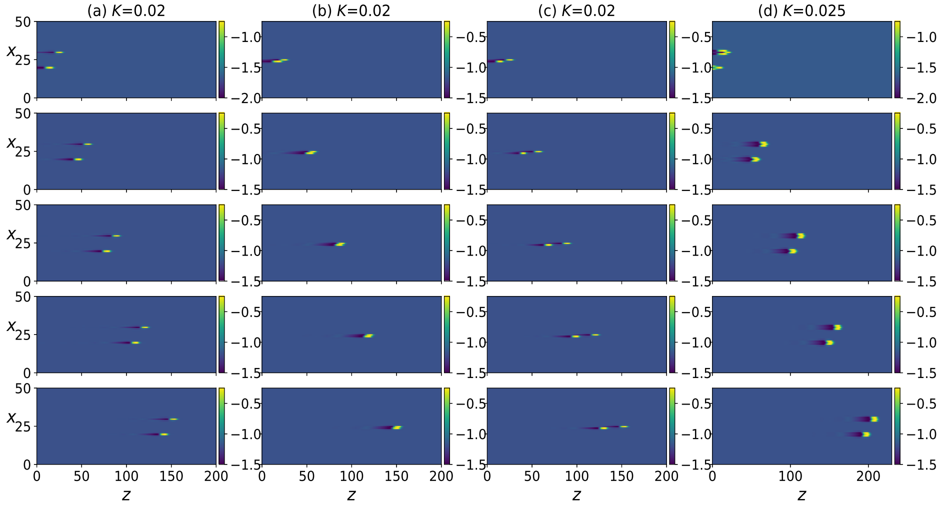

Figure 2: **(SI Fig.2) Numerical simulation results for Eqs.15 equivalent to results in Fig.3 for Eqs.13.** The color bar indicates the value of v, the x variable indicates the axon number. (a) $K = 0.02$, axons number 30 and 20 are stimulated at $t = 0$ and $t = 10$, respectively and the panel rows from top to bottom correspond to $t = 500, 1100, 1700, 2300, 2900$. (b) same as in (a) but with axons number 25 and 24 stimulated at $t = 0$ and $t = 9$, respectively. (c) same as in (b) but with stimulation at $t = 0$ and $t = 10$. (d) same as in (a) but with $K = 0.025$ and panels show $t = 500, 1300, 2200, 3100, 3999$.

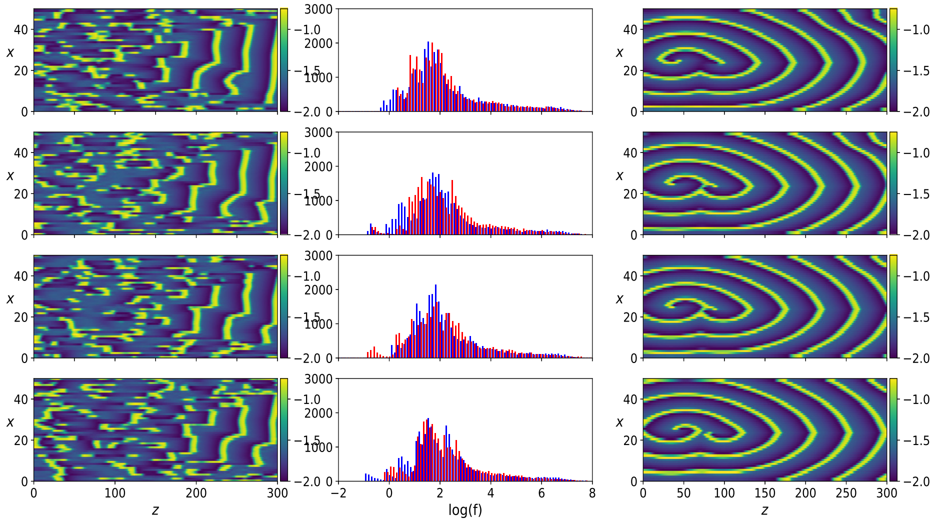

Figure 3: **(SI Fig.3) Comparison of the spatial patterns of the two systems, Eqs.13 and Eqs.15.** Numerical simulation of Eqs.13 for $R = 0.15$ (left) and of Eqs.15 for $K = 0.04$ (right). The middle column shows a comparison of the spectrograms of the spatial discrete Fourier transform of the response of the two systems at specific times: Eqs.13 (red), Eqs.15 (blue).The panel rows from top to bottom correspond to $t = 8000, 8600, 9350, 9950$.

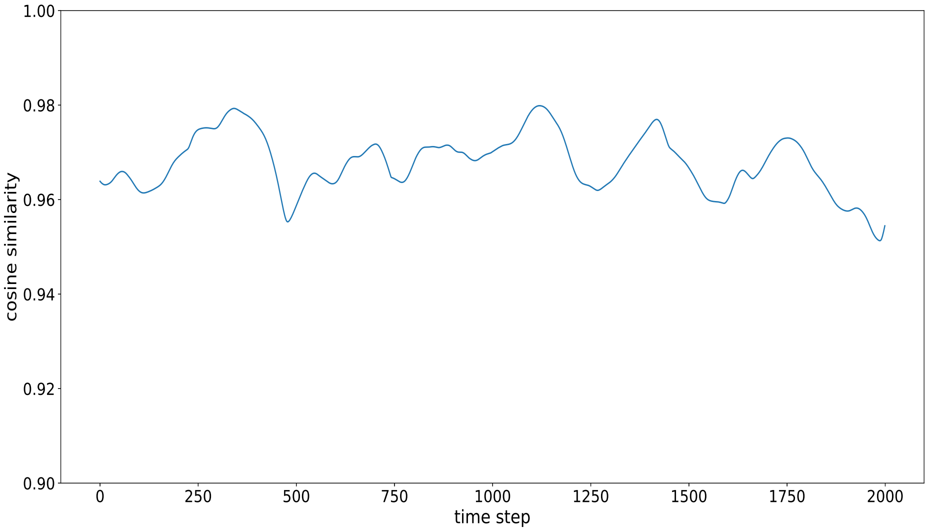

Figure 4: **(SI Fig.4) Cosine similarity between the discrete Fourier transform of the spatial patterns of the two systems**. The cosine similarity between the spatial discrete Fourier transform of the spatial patterns of the solutions of Eqs.13 and Eqs.15 with $R = 0.15$ and $K = 0.04$, respectively.

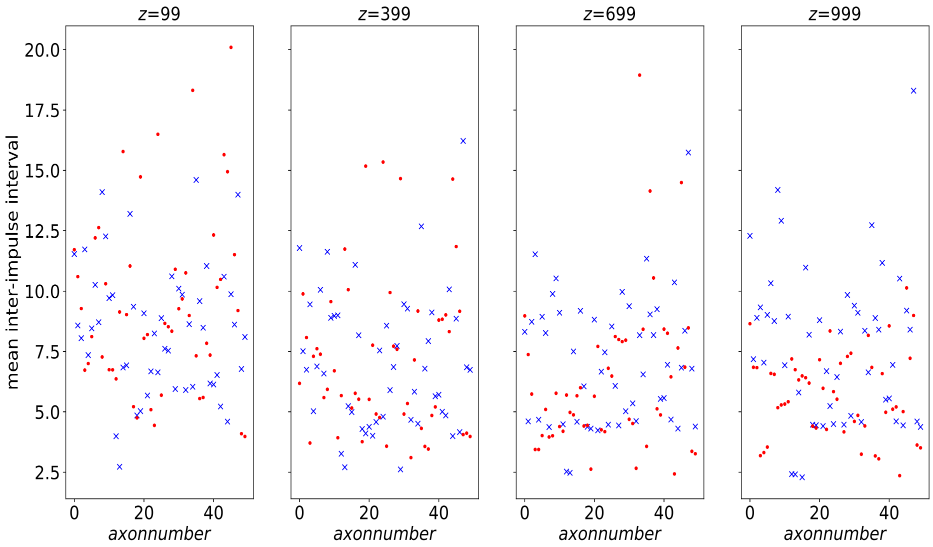

Figure 5: **(SI Fig.5) mISI for each axon at different z locations for Eqs.13.** For the simulation in Fig.7(a) $R = 1000$ (blue x) and (b) $R = 0.7$ (red dot).

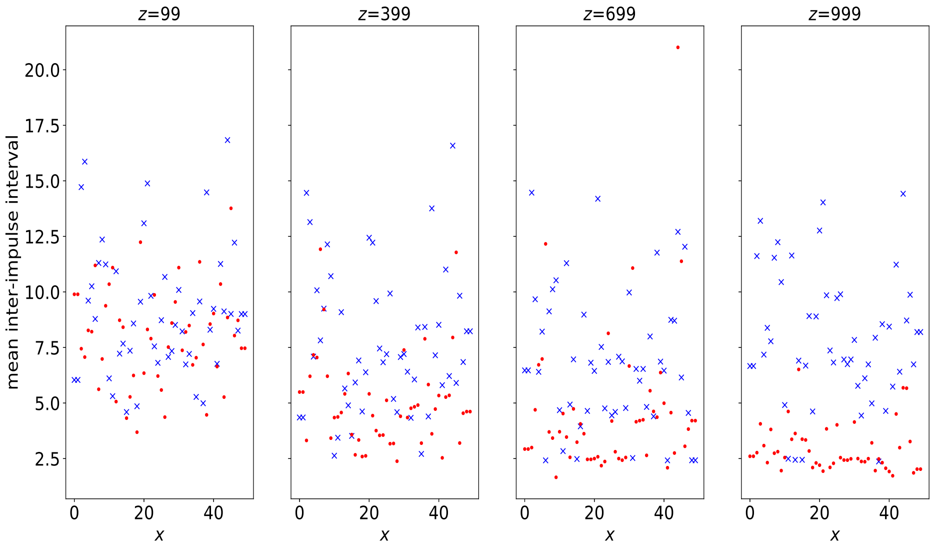

Figure 6: **(SI Fig.6) mISI for each axon at different z locations for Eqs.15.** For the simulation in Fig.7(c) $K = 0$ (blue x) and (d) $K = 0.025$ (red dot).
